# A Machine Vision-Based Method for Monitoring Broiler Chicken Floor Distribution

**DOI:** 10.3390/s20113179

**Published:** 2020-06-03

**Authors:** Yangyang Guo, Lilong Chai, Samuel E. Aggrey, Adelumola Oladeinde, Jasmine Johnson, Gregory Zock

**Affiliations:** 1 Department of Poultry Science, College of Agricultural & Environmental Sciences, University of Georgia, Athens, GA 30602, USA; yangyang.guo@uga.edu (Y.G.); saggrey@uga.edu (S.E.A.); ade.oladeinde@ars.usda.gov (A.O.); jcj36120@uga.edu (J.J.); gregzock@uga.edu (G.Z.); 2 College of Mechanical and Electronic Engineering, Northwest A&F University, Yangling, Shaanxi 712100, China; 3 U.S. National Poultry Research Center, USDA-ARS, Athens, GA 30605, USA

**Keywords:** broiler chicken, health and welfare, animal behaviors, precision farming

## Abstract

The proper spatial distribution of chickens is an indication of a healthy flock. Routine inspections of broiler chicken floor distribution are done manually in commercial grow-out houses every day, which is labor intensive and time consuming. This task requires an efficient and automatic system that can monitor the chicken’s floor distributions. In the current study, a machine vision-based method was developed and tested in an experimental broiler house. For the new method to recognize bird distribution in the images, the pen floor was virtually defined/divided into drinking, feeding, and rest/exercise zones. As broiler chickens grew, the images collected each day were analyzed separately to avoid biases caused by changes of body weight/size over time. About 7000 chicken areas/profiles were extracted from images collected from 18 to 35 days of age to build a BP neural network model for floor distribution analysis, and another 200 images were used to validate the model. The results showed that the identification accuracies of bird distribution in the drinking and feeding zones were 0.9419 and 0.9544, respectively. The correlation coefficient (R), mean square error (MSE), and mean absolute error (MAE) of the BP model were 0.996, 0.038, and 0.178, respectively, in our analysis of broiler distribution. Missed detections were mainly caused by interference with the equipment (e.g., the feeder hanging chain and water line); studies are ongoing to address these issues. This study provides the basis for devising a real-time evaluation tool to detect broiler chicken floor distribution and behavior in commercial facilities.

## 1. Introduction

In commercial poultry houses, animal floor uniformity and distribution in drinking, feeding, and resting zones are critical for evaluating flock production, animal health, and wellbeing. The proper distribution of chickens is an indication of a healthy flock. Currently, daily routine inspections of broiler flock distributions are done manually in commercial grow-out houses, which is labor intensive and time consuming. This task requires an efficient system that can monitor chicken floor distribution and behavior automatically, to provide information for the early detection of potential problems [1,2,3]. 

Noncontact and nondestructive monitoring methods such as the machine vision-based technology (MVT) have been suggested and tested to monitor poultry and livestock behavior and for individual identification [4,5,6,7,8,9]. MVT has also been used to evaluate welfare status (e.g., lameness, estrus, pecking, etc.) [10,11,12,13], and for body size or weight assessments [14,15]. For poultry housing, different versions of MVT have been tested for the identification of specific behaviors under given scenarios (e.g., feeding and drinking as affected by environmental factors or enrichments) and general group behavior (e.g., activity index and locomotion) with or without assistance from other sensors (e.g., Radio-frequency identification and accelerometers) [16,17,18,19,20,21]. However, most existing procedures have limitations or high levels of uncertainty in monitoring group chicken behavior and distribution in the different feeding, drinking, and resting zones due to higher animal density (>10,000 broiler chickens in a commercial facility) compared to other animal (e.g., cattle and swine) facilities [7,8,22]. The “optical flow” method for measuring broiler welfare based on optical flow statistics of flock movements recorded on video [19,22,23], and the “eYeNamic” system for gait score monitoring in broiler houses [17,24], are the most common vision-based methods. These systems represent the first proof of concept for broiler welfare evaluation via computer or machine vision-based methods. However, there is no method, to our knowledge, with a high level of efficiency and accuracy for monitoring group bird behavior or for tracking individual birds in a commercial broiler house setting. 

The back propagation (BP) neural network algorithm is a multilayer feedforward network trained according to the error back propagation algorithm [25]. This algorithm is one of the most widely applied neural network models. BP networks can be used to learn and store a great deal of mapping relation data via an input-output model; there is no need to disclose in advance the mathematical equation that describes these mapping relations. In recent years, a BP neural network algorithm was tested and showed a high level of accuracy in monitoring the production and behavior of large animals, e.g., pigs’ pen floor behavior recognition, rating, and body weight prediction [26,27,28,29]. Applying this method to monitor poultry requires modifications to the algorithm and training with a large number of animal images reflecting changes in body size. 

The objectives of this study were to: 1) develop a machine vision-based method for monitoring broiler chicken floor distribution (i.e., real-time number of birds in the drinking, feeding, and resting zones); 2) train the BP neural network model with broiler chicken images collected at different ages; and 3) test the new machine vision-based method in terms of its ability to identify the distribution of broiler chickens in the feeding and drinking zones of a research poultry facility. 

## 2. Materials and Methods

### 2.1. Experimental Setup and Data Collection

**Figure 1:**
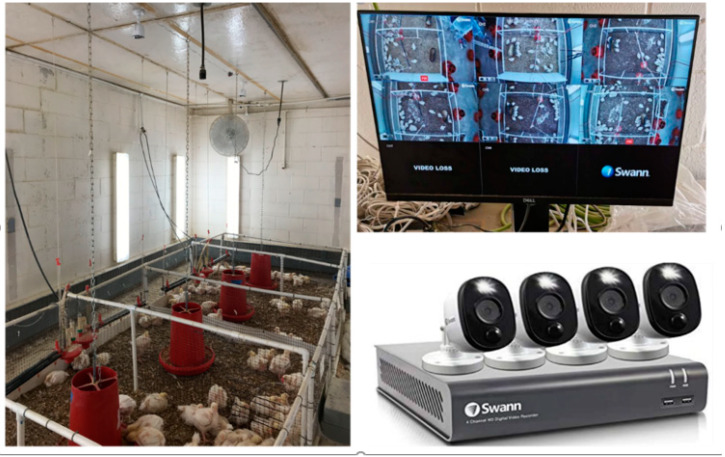
Experimental setup for broiler chicken image data collection.

This study was conducted in an experimental facility (Figure 1) at the Poultry Research Center at the University of Georgia, Athens, GA, USA. Six identical pens measuring 1.84 L × 1.16 W m were used to raise Cobb 500 broiler chickens (21 broilers per pen) from d1 to d49 (from November 26, 2019 to January 14, 2020). Each pen was monitored with a high definition (HD) camera (PRO-1080MSFB, Swann Communications, Santa Fe Springs, CA) mounted on the ceiling (2.5 m above floor) to capture video (15 frame/s with the resolution of 1440 × 1080 pixels). Videos were saved as avi files in a video recorder (DVR-4580, Swann Communications, Santa Fe Springs, CA) (Figure 1). The collected videos were transferred to a data station in an office every three day. The files were later converted to images using MATLAB-R2019b. 

**Figure 2:**
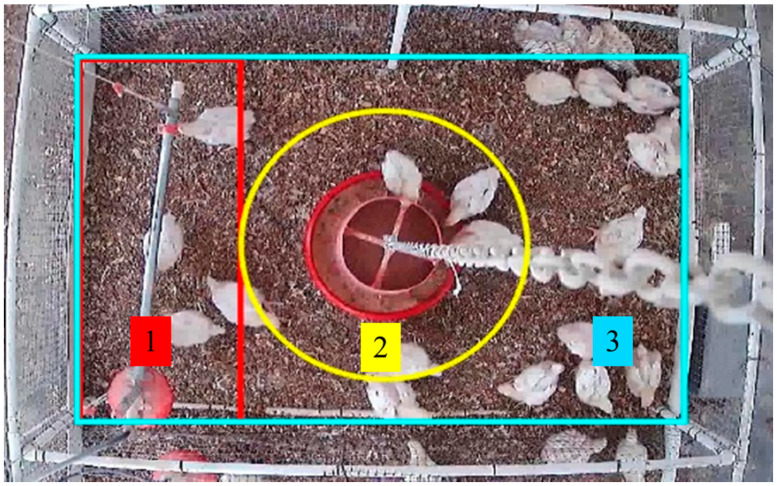
A top view of a pen and zone definition. The red box (1) represents the drinking area: the center of the nipple drinker is the center of the drinking area and its width is defined as one body length of a three-week-old broiler chicken; the yellow circle (2) represents the feeding area: the center of the tube feeder is the feeding area center; the radius of feeding zone is the tube feeder radius plus the body length of a three-week-old broiler chicken; and the cyan box (3) represents the overall detection area of the pen, so any area not included in drinking and feeding zones will be considered as the rest/exercise zone.

For the machine-vision based method to recognize the bird distributions in the images, the pen floor was divided virtually into drinking, feeding, and rest/exercise zones (Figure 2). Broilers were raised antibiotic-free on reused litter made of pine shavings. Husbandry and management (e.g., feeding, drinking, lighting, bedding, and house air temperature) followed the US industry standard protocols and approval was obtained from the Institutional Animal Care and Use Committee (IACUC) at the University of Georgia. 

### 2.2. Method for Target (Chicken) Detection 

**Figure 3:**
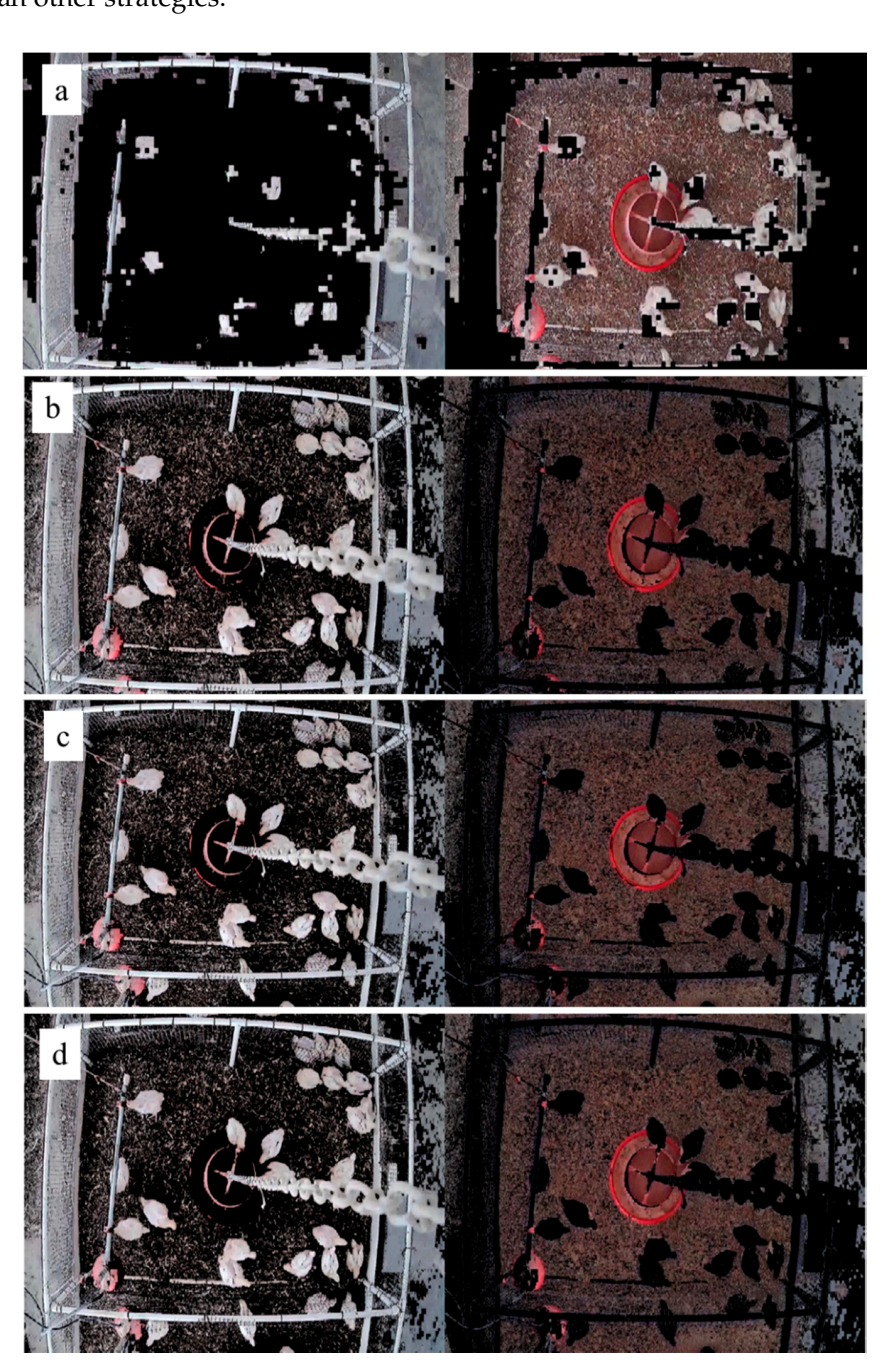
Comparison between different color spaces ((**a**) LAB, (**b**) RG, (**c**) RB, and (**d**) GB) in the classification process.

The target detection method in the current study was developed based on the color space classification in the MATLAB tool box (MATLAB-R2019b, The MathWorks, Inc., Natick, MA). The MATLAB tool box provides a number of different color space classification choices such as L* a* b* (LAB) and RGB (Red, Green, Blue) for RG, RB, and GB. We compared the visualization effect of different color space classification methods (Figure 3). The GB method was used in this study, as it (Figure 3d) had higher classification and visualization efficiencies (e.g., processing time and target extraction) than other strategies. 

**Figure 4:**
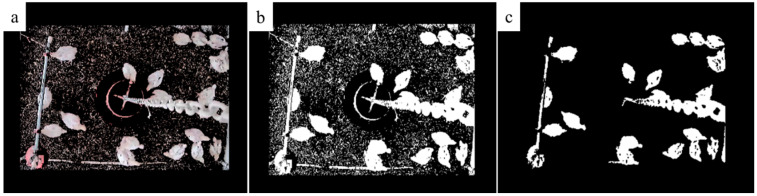
The image processing steps. (**a**) Nontarget area removal; (**b**) Generation of binary image; (**c**) Morphological corrosion operation and background removal.

Two-dimensional Otsu processing was applied to convert the original images into binary images [30]. Figure 2 (an original image) was used as an example to show image processing steps of removing the nontarget area (Figure 4a), the generation of binary image (Figure 4b), and a further process with morphological corrosion to remove the background (Figure 4c). It can be observed that the nipple drinker pipe and the hanging chain of the tube feeder were blocking the top view images of the chickens on the floor. 

In addition, the current method (i.e., the integration of GB color space and two-dimensional Otsu processing) was compared, in terms of its image processing speed and visualization efficiency, to K-means [31] and Fuzzy C-Means (FCM) [32], two widely used classical clustering algorithms to identify static or mobile targets. The K-means algorithm takes *k* as the parameter to divide *n* objects into *k* clusters, such that the cluster has a high degree of similarity, while the similarities between clusters are low [31]. FCM is a clustering method based on fuzzy sets which determines the subordination of each data point to a center by membership degree [32]. 

### 2.3. Method for Counting Broiler Chickens

The top view area-based animal recognition method was used to determine the number of broilers in different zones on the floor [33]. For a machine vision-based method, recognizing chickens and their numbers in an image is based on chicken profile and specific area size, because they tend to congregate together. The images collected each day were analyzed separately to avoid biases in each of the top view images caused by changes in the body weight/size over time. Different chicken profiles were randomly selected from images collected each day. The area of each chicken in the image was quantified first, then the average was used as the reference area of the day to estimate the number of chickens on the house floor, as expressed in Equation (1).

(1) si¯=sis , 1≤i≤n

where $\bar{s_{i}}$ is the area value of the *i*^th^ area after normalization, $s_{i}$ is the area value of the *i*^th^ area in the image, *s* is the reference value of a single broiler area, and *n* is the number of areas detected in the image.

To determine the chicken number in each zone, the specific BP neural network algorithm was developed and applied by referring existing methods [29,34]. A BP network is a typical supervised neural network classifier which performs the function of linear or nonlinear mapping from input to output to automatically extract reasonable solving rules through learning; it has certain generalization abilities [25]. In the current study, the newly modified BP neural network model comprised an input layer (area value of a chicken), a hidden layer, an output layer (the number of chicken), and a node connection between the layers. On the MATLAB-R2019b software platform (MathWorks, Natick, MA), the Feedforwardnet function was used to build a BP neural network.

We manually selected high quality images collected during d18–d35 (1926 images in total) to train the model to identify broiler chickens in the drinking and feeding zones. Then, an additional 196 randomly selected images between d18 and d35 were used for verification. The quantification method is expressed in Equations (2)–(6). According to different zones defined in Figure 2, chickens were considered to be in the drinking or feeding zone when more than 50% of their body was quantified in the zone.

(2) $$D_{num}=\overset{n}{\underset{i=1}{\sum }}round\left(\frac{A_{di}}{A_{sc}}\times N_{i}\right)$$

(3) $$F_{num}=\sum_{j=1}^{m}round\left(\frac{A_{fj}}{A_{sc}}\times N_{j}\right)$$

(4) $$R_{accuracy}=\frac{T_{num}}{T_{truenum}}$$

(5) $$R_{miss}=\frac{T_{miss}}{T_{truenum}}$$

(6) $$R_{false}=\frac{T_{false}}{T_{truenum}}$$

where *D_num_* is the number of broiler chickens detected in the drinking zone, *n* is the number of extracted areas containing chicken/chickens in the drinking zone, *A_di_* is the size of the i^th^ extracted area containing chicken/chickens in the drinking zone, *A_cs_* is the standardized area size of a chicken in the image, *N_i_* is the number of broiler chicken associated area detected in the *A_di_*, *F_num_* is the number of broiler chickens detected in the feeding zone, *m* is the number of extracted areas containing chicken/chickens in the feeding zone, *A_fj_* is the size of the j-th extracted area containing chicken/chickens in the feeding zone, *N_j_* is the number of broiler chicken associated area detected in the *A_dj_*, *R_accuracy_* is the accuracy rate of the number of broilers in the drinking area or feeding area, *T_num_* is the number of broiler chickens detected automatically within the drinking or feeding zone, *T_truenum_* is the true number of broiler chickens in the drinking or feeding zone, *R_miss_* is the missed detection rate, *T_miss_* is the number of missed detections, *R_false_* is the false detection rate, and *T_false_* is the number of false detections.

### 2.4. Evaluation Criteria and Statistical Analysis

In order to measure the calculated deviation (i.e., difference between true and predicted results) and test the new BP model in chicken number determination, three test criteria, i.e., correlation coefficient R, mean square error (MSE), and mean absolute error (MAE), were applied [29].

(7) $$R=\sqrt{1-\overset{n}{\underset{i=1}{\sum }}\frac{{\left(y_{i}-\hat{y_{i}}\right)}^{2}}{{\left(y_{i}-\bar{y_{i}}\right)}^{2}}}$$

(8) $$\mathrm{MSE}\;=\frac{\sum_{i=1}^{n}{\left(y_{i}-\hat{y_{i}}\right)}^{2}}{n}$$

(9) $$\mathrm{MAE}\;=\frac{1}{n}\sum_{i=1}^{n}\frac{\left|y_{i}-\hat{y_{i}}\right|}{y_{i}}$$

where, $y_{i}$ is the actual number of broilers, $\hat{y_{i}}$ is the number obtained by model fitting, $\bar{y_{i}}$ is the average of the actual number, and *n* is the number of samples.

A one-way ANOVA (MATLAB-R2019b) was used to test if there were significant differences in the detection speed of broiler chickens on the same image between the method developed in the current study (i.e., integration of GB Color Space and two-dimensional Otsu processing), K-means, and FCM. The effect was considered to be significant when the *p*-value was less than 0.05.

## 3. Results and Discussions

### 3.1. Individual Chicken Identification

**Figure 5:**
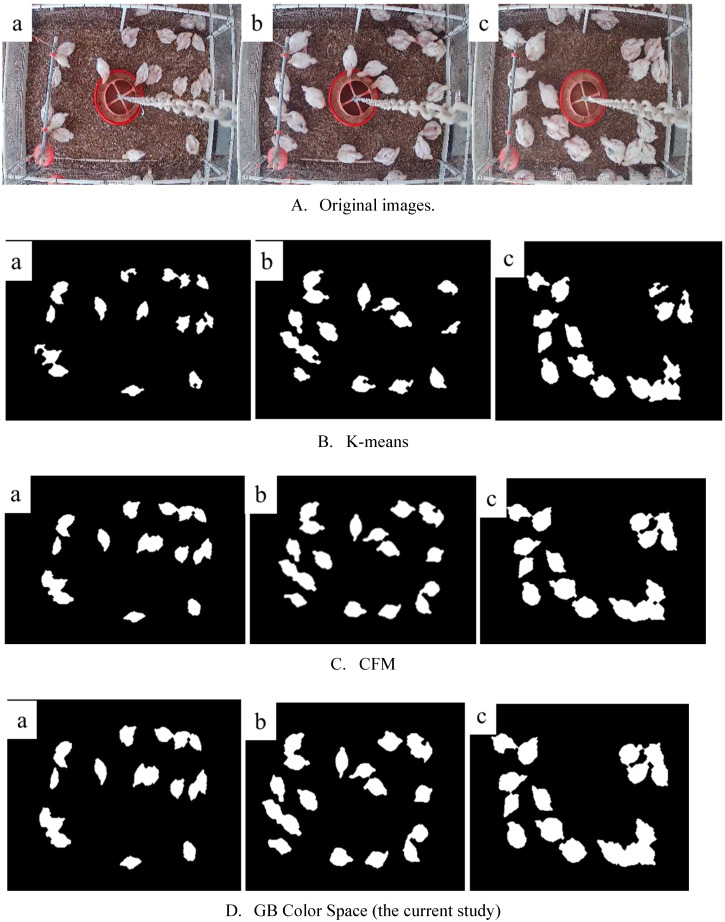
The visualization efficiency of three different methods (**a**, **b**, and **c** represent images taken on d18, d24 and d30, respectively).

Images collected on d18, d24, and d30 were randomly selected (30 images) to test the GB color space classification for chicken identification. Figure 5 shows the identification results (i.e., profile extraction of chicken/chickens) of the current method and the comparison with two other existing methods, i.e., K-means and FCM. The current method extracted individual animals from original images with a visualization efficiency (e.g., clearness and completeness of chicken areas) similar to FCM method, but a better visualization effect than K-means method (*p* < 0.001). However, the current method required less time (clustering speed) than FCM (*p < 0.001*) (Table 1). According to Figure 5B, some target areas (i.e., chicken profiles) were lost during imaging processing with the K-means method. 

### 3.2. Broiler Chicken BP Model Building/Training Results

**Figure 6:**
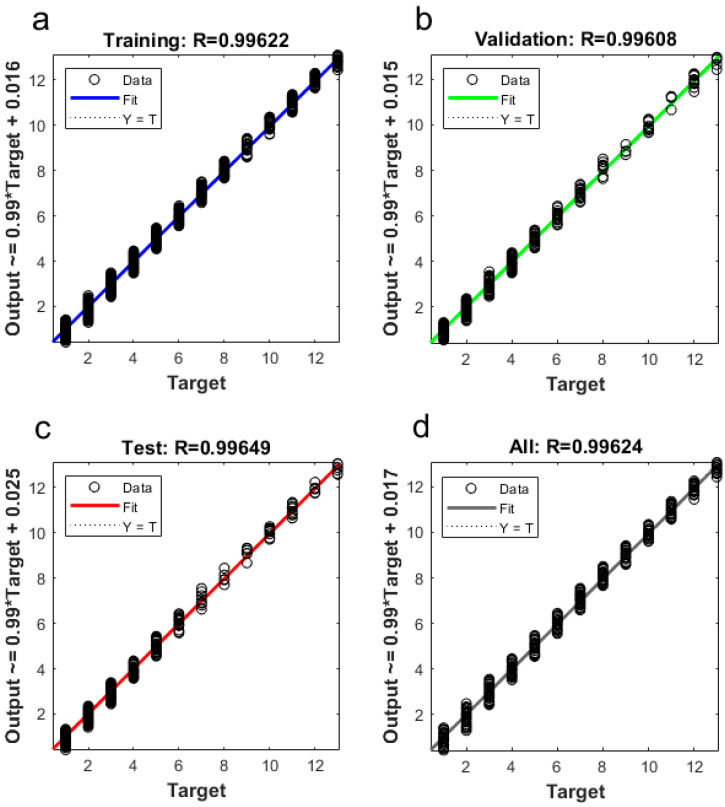
Input–output correlation in the newly developed BP neural network model for identifying broiler chicken floor distribution (**a**,**b**,**c**, and **d** correspond to the training set, validation set, test set and overall results, respectively. The horizontal axis *Target* is the actual number of chickens; and the vertical axis *Output* is the number of BP model output; “O” represents the input data of the model; “Fit” is the fitting relationship between input and output; “Y = T” means the training output value equal to the target value).

From the images collected from d18 to d35 of chicken age, the video segments (6–8 min) in each hour were used to train the BP neural network model. Finally, about 1926 images were used to obtain 19,988 target chicken areas. Among these areas, about 6896 target areas qualified as good (i.e., area without overcrowding or occlusion) for building/training the broiler chicken BP network model. The ratios of vectors for model training, validation, and testing were 0.70, 0.15, and 0.15, respectively. The newly trained BP model had an MSE of 0.038, MAE of 0.178, and R of 0.996 (Figure 6). 

### 3.3. Chicken Distribution Identification with BP Model

#### 3.3.1. Total Chicken Numbers Identification

**Figure 7:**
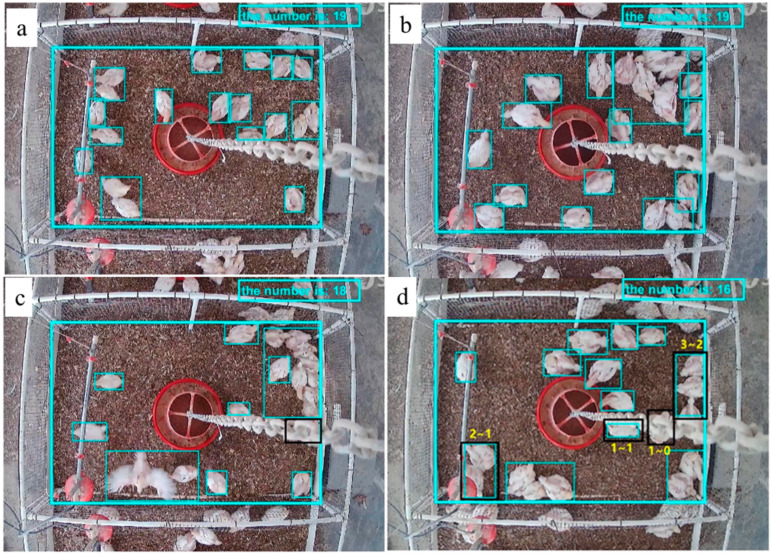
Number of chickens identified and their distribution determined with the newly developed BP model (chickens were three weeks of old in (**a**), (**b**) and 4 weeks old in (**c**), (**d**); cyan rectangles represent target extraction zone by BP method, and back rectangles represent missed target area (chicken); yellow numbers indicate true chicken number and behind indicate BP model recognized chicken).

Figure 7 shows the number of broiler chickens automatically counted by the BP model. Originally, there were 21 chickens, but two were sampled from each pen for health evaluation, leaving 19 in each pen. The BP model could identify the chicken distribution at different ages correctly by quantifying the number of chickens in each zone (Figure 7a,b). However, broiler chickens could be blocked by equipment including the feeder hanging chains and the water lines from the top view images (Figure 7c,d).

The interference could be fixed after image background processing only if a part of a body was blocked (e.g., a chicken blocked by feeder chain was still recognizable, Figure 7a). Sometimes, the issue cannot be fixed if the chicken is mostly obscured (>50% area) (e.g., one chicken was missed in Figure 7b,d, respectively). The interference issues caused by physical barriers can be solved by using multiple cameras or a mobile camera. In addition, broiler chickens were observed spreading their wings and crowded together during data collection, which affected the accuracy of chicken counting (Figure 7c). For instance, there were only two broiler chickens in the bottom left area of Figure 7c, with one chicken spreading its wings, but this was misidentified as five chickens due to the expansion of the target area. Therefore, there is a need to improve the chicken identification efficiency of this model before counting can be applied in a commercial setting, where thousands of birds are usually crowded together.

#### 3.3.2. Chicken Distribution Identification in Drinking and Feeding Zones

**Figure 8:**
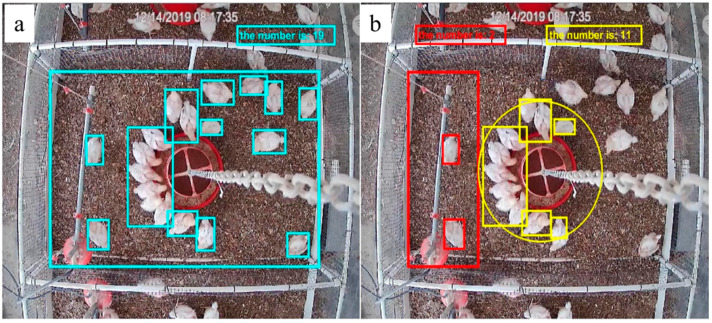
Broiler chicken distribution in feeding and drinking zones as identified by the BP model. (**a**) total chicken tracked and identified; (**b**) chicken distribution in drinking and feeding zones. The big red rectangle is the drinking zone, and the small red rectangles in this zone indicate the detected broiler chickens. The yellow circle is the feeding area, and the yellow rectangles in the zone indicate detected broiler chickens.

Figure 8 shows the distribution of broiler chickens in feeding and drinking zones identified by the BP model. For the same image, the model analyzed the total number of chickens in the pen first (Figure 8a), and then quantified their distribution in each zone (Figure 8b). From Figure 8b, we can ascertain that there were two birds in the drinking zone and 11 in the feeding zone. The rest (19 − 2 − 11 = 6) were considered to be in rest zone. 

Table 2 shows details of the distribution analysis of 196 images randomly selected from images collected between d18-d35 to verify the BP method. The identification accuracy rates for broiler chicken distribution in the drinking and feeding zones were 0.9419 and 0.9544, respectively. The missed detection rates were 0.0626 and 0.0498, respectively; this was primarily caused by chicken crowding behavior and occlusion problems from the feeder hanging chains and water system (Figure 8). The false detection rates were 0.0045 and 0.0037, respectively. The current method was developed using healthy chickens. However, in the future, the model will be applied to chickens raised under several conditions (e.g., diseases, heat stress, etc.) in order to develop real-time welfare-status evaluation ability. 

It is a technical challenge to apply only machine vision-based methods to judge individual eating or drinking behavior in commercial broilers houses with thousands of chickens. In the current study, we focused on the floor distribution patterns (i.e., real-time counts of bird numbers in the feeding and drinking zones), as this is technically quantifiable and the information is correlated to animal health and welfare, as birds with underlying conditions such as lameness or high gait score tend to be less active and stay closer to feeders/drinkers due to their limited mobility [10,13]. Our method is different from most existing methods. The “eYeNamic” system, for instance, evaluates a gait score based on a group activity index quantified according to pixel changes in continuously recorded photos/videos. The broiler house is a physically challenging environment for vision-based monitoring due to equipment interference and dust levels [35,36], and usually leads to poor image quality and results. In the long-term, we plan to develop a mobile imaging system equipped with a global positioning system (GPS) to track individual birds for their welfare/health based on their distribution patterns. 

## 4. Conclusions

A machine vision-based method was developed and tested to identify broiler chicken floor distributions, including the total number of chickens on the house floor and their distribution in drinking and feeding zones. The following observations and conclusions were made from the current study:

(1) Advanced image processing techniques of GB color space and two-dimensional Otsu processing were integrated for image processing, which has a faster clustering speed than most existing methods (e.g., K-means and FCM) (*p* < 0.001);
(2) The BP neutral network model developed to count the total number of birds on the floor and their distribution in feeding and drinking zones had a correlation coefficient (R), mean square error (MSE), and mean absolute error (MAE) of 0.996, 0.038, and 0.178, respectively;
(3) The machine vison-based method was tested with an accuracy rate of 0.9419 and 0.9544, respectively. The missed detections were primarily caused by facility interferences such as feeder hanging chains and water lines in the chicken images. These issues can be solved by using multiple cameras or a mobile imaging operation.

Work is ongoing to identify chicken head posture and distance from feeder, drinker and litter floor in order to evaluate real-time feeding, drinking, and foraging behaviors. The current findings provide the basis for the development of an automatic system to monitor poultry floor distributions and behavior in a commercial production system.

## Figures and Tables

**Table 1:** Comparison between different methods in processing (clustering) speed for 30 images on different days.

Method	Images Clustering Running Time (s, Mean ± SD, n = 30)		
	d18	d24	d30
K-means	5.17 ± 0.69	5.04 ± 0.66	5.31 ± 0.87
FCM ^1^	16.79 ± 1.26	14.93 ± 0.86	12.99 ± 0.81
This study ^2^	0.24 ± 0.04	0.26 ± 0.03	0.24 ± 0.04

^1^ FCM-Fuzzy C-Means. ^2^ The integration of GB color space and two-dimensional Otsu processing.

**Table 2:** Test of the BP model on 196 images on identification of chicken distribution in the feeding/drinking zones.

Zone	True Chickens ^1^	Detected Chickens ^2^	Missed Detections [3]			False Detections	*R_ac_*	*R_miss_*	*R_false_*
			Crowding	Occlusion	Others				
**Drinking**	671	632	8	32	2	3	0.9419	0.0626	0.0045
**Feeding**	823	785	8	26	7	3	0.9544	0.0498	0.0037

^1^ True chicken numbers were obtained manually by looking at 196 images; ^2^ Detected chicken numbers were obtained by applying the newly developed BP model to analyze 196 images automatically; ^3^ Missed detections were caused by multiple factors such as the crowding of chickens and occlusion by equipment such as feeder hanging chains and water lines; *R_ac_*-identification accuracy rate of the number of broilers in the drinking/feeding zone, *R_miss_* -missed detection rate, and *R_false_*-false detection rate.
